# A Hyperthermophilic Argonaute From *Ferroglobus placidus* With Specificity on Guide Binding Pattern

**DOI:** 10.3389/fmicb.2021.654345

**Published:** 2021-06-09

**Authors:** Xiang Guo, Yingying Sun, Liuqing Chen, Fei Huang, Qian Liu, Yan Feng

**Affiliations:** ^1^State Key Laboratory of Microbial Metabolism, School of Life Sciences and Biotechnology, Shanghai Jiao Tong University, Shanghai, China; ^2^Shenzhen Institutes of Advanced Technology, Chinese Academy of Sciences, Shenzhen, China

**Keywords:** Argonaute, thermophilic archaea, *Ferroglobus placidus*, endonuclease, structural analysis

## Abstract

Argonaute proteins (Agos) from thermophilic archaea are involved in several important processes, such as host defense and DNA replication. The catalytic mechanism of Ago from different microbes with great diversity and genome editing potential is attracting increasing attention. Here, we describe an Argonaute from hyperthermophilic *Ferroglobus placidus* (*Fp*Ago), with a typical DNA-guided DNA endonuclease activity but adopted with only a short guide 15–20 nt length rather than a broad guide selectivity for reported Agos. *Fp*Ago performed the precise cleavage of phosphodiester bonds between 10 and 11 nt on the target strand (counting from the guide strand) guided strictly by 5′-phosphorylated DNA at temperatures ranging from 75 to 99°C. The cleavage activity was regulated by the divalent cations Mn^2+^, Mg^2+^, Co^2+^, and Ni^2+^. In addition, *Fp*Ago possesses guide/target mismatch tolerance in the seed region but is sensitive to mismatches in the 3′-guide region. Notably, the EMSA assay revealed that the *Fp*Ago-guide-target ternary complex exhibited a stronger binding affinity for short 15 and 16 nt guide DNAs than longer guides. Moreover, we performed structural modeling analyses that implied the unique PAZ domain of *Fp*Ago for 3′-guide recognition and binding to affect guide length specificity. This study broadens our understanding of thermophilic Agos and paves the way for their use in DNA manipulation.

## Introduction

Argonaute proteins (Agos) are widely present in all domains of life. Eukaryotic Argonaute proteins (eAgos) are the best studied and have been demonstrated to be involved in RNA interference using single-stranded RNA (ssRNA) guides (Ketting, 2011; Schirle and MacRae, 2012; Swarts et al., 2014b; Hegge et al., 2018). Recent studies have revealed that prokaryotic Argonaute proteins (pAgos) participate in host defense against invasive genetic elements and function in completing DNA replication (Swarts et al., 2015; Jolly et al., 2020; Kuzmenko et al., 2020). Unlike eAgos, pAgos exhibit high diversity in catalytic and biological functions, from the guide/target nuclei acid type, binding/cleavage activity, to pathways involved (Hegge et al., 2018). Due to its programmable endonuclease activity, researchers are interested in mining new pAgos and extending pools for genome-editing applications.

To date, only a few pAgos derived from thermophilic archaea and mesophilic bacteria have been well characterized (Swarts et al., 2014a, 2015; Kaya et al., 2016; Zander et al., 2017; Cao et al., 2019; Chong et al., 2019; Hegge et al., 2019; Kuzmenko et al., 2019; Kim et al., 2020; Olina et al., 2020; Liu et al., 2021b). Some Agos can act as programmable endonucleases to cleave targets between nucleotides 10 and 11 using a divalent cation to catalyze the hydrolysis of the phosphodiester bond that lies across nucleotides 10 and 11 of the base-pairing guide (Swarts et al., 2015; Jolly et al., 2020; Kuzmenko et al., 2020). This guide-directed specific cleavage is similar to the clustered regularly interspaced short palindromic repeat (CRISPR)-associated protein (Cas) system (Marraffini and Sontheimer, 2010; Jinek et al., 2012; Taly et al., 2013; Barrangou and Marraffini, 2014; Zhang, 2019). Compared to its analogous CRISPR-Cas nucleases, the precise recognition capacity of Ago is mediated by guide-target pairing without requiring the presence of any specific motifs of the target sequence, which makes Ago target any DNA fragment of interest. Furthermore, short DNA or RNA oligonucleotides, usually at 21 nt, utilized by Agos as guide molecules, are much easier and cheaper to synthesize than longer RNA guides required for Cas nucleases. Due to the unparalleled simplicity, programmability, and specific cleavage activity, Agos hold powerful potential for genetic manipulation. An artificial restriction enzyme platform based on *Pyrococcus furiosus* Argonaute (*Pf*Ago) was developed to recognize and cleave DNA sequences at virtually any arbitrary site (Enghiad and Zhao, 2017). Furthermore, thermophilic pAgos have been recently used as programmable tools for specific target detection and rare single nucleotide variant enrichment in molecular diagnosis applications (He et al., 2019; Xun et al., 2019; Liu et al., 2021a; Song et al., 2020).

Due to the increasing importance of Agos in nucleic acid manipulation, we set out to identify novel Agos that possess desired biochemical properties with high specificity. In this study, an Ago from the hyperthermophilic archaeon *Ferroglobus placidus* (*Fp*Ago) was biochemically characterized. The results demonstrated that *Fp*Ago functions as a DNA-guided DNA endonuclease and shows a specific preference for guide utilization on the 3′-terminal. These new enzyme properties would provide new catalytic applications in the manipulation of DNA.

## Materials and Methods

### Sequence Analysis

To discover new potential pAgos candidates, BLAST, based on *Pf*Ago amino acid sequences, was performed in the NCBI database, and the sequences were selected for phylogenetic analysis using MEGA 7.0.

### *Fp*Ago Expression and Purification

The His-tagged (N-terminal) *Fp*Ago gene with optimized codons was synthesized and cloned into the pET-28a (+) plasmid to yield the expression plasmid pET-28a (+)-*Fp*Ago by Sangon Biotech (Shanghai, China). The expression plasmid was transformed into *E. coli* BL21 (DE3) host strain. *Fp*Ago was overexpressed and purified as previously described (Chong et al., 2019). The expression of recombinant *Fp*Ago was induced by 0.5 mM isopropyl-beta-D-thiogalactopyranoside (IPTG) at 20°C for 20 h, and the resulting protein was purified using an Ni-NTA affinity column. The purity of *Fp*Ago was determined using SDS-PAGE. The protein concentration was determined using the BCA method. The purified *Fp*Ago was stored in 15% (v/v) glycerol at −80°C until further use.

### Enzymatic Activity Assay

For the standard activity assays, a total of 400 nM *Fp*Ago was mixed with synthetic 2,000 nM guides, and 800 nM targets in reaction buffer (15 mM Tris/HCl pH 8, 250 mM NaCl) supplemented with 0.5 mM Mn^2+^ and incubated for 15 min at 95°C, except when otherwise indicated. The optimal temperature for *Fp*Ago activity was assessed at temperatures ranging from 65 to 99°C. The effects of divalent metal ions on *Fp*Ago activity were measured in the presence of Ca^2+^, Co^2+^, Cu^2+^, Mg^2+^, Mn^2+^, Zn^2+^, Ni^2+^, or EDTA. The effects of NaCl concentration on *Fp*Ago activity were investigated using various NaCl concentrations (50–2000 mM). The preference for the 5′- end nucleotide of guide DNA (gDNA) was tested by providing DNA guides containing a 5′-dC, dT, dA, or dG. The influence of 5′- modifications of gDNA on *Fp*Ago activity was assessed by different chemical modifications on the 5′-terminal (-P, -OH, -NH2, -SH, -Biotin, -Cy3, -Cy5, -BHQ1, -ROX, -FAM, -VIC). The optimal gDNA length adopted by *Fp*Ago was measured in the presence of 11–21 nt gDNA. The minimum length of the target required for *Fp*Ago catalytic activity was determined in the presence of 14–60 nt target DNA. A single mismatch was introduced in the gDNA from positions 2–15, respectively, to test the guide-target mismatch tolerance. All cleavage experiments were performed in triplicates. Reactions were stopped by rapid cooling to 4°C, followed by the addition of a loading buffer (Sangon Biotech, Shanghai, China) at a 1:1 ratio (v/v). The samples were then resolved on a 16% denaturing polyacrylamide gel. The gels were stained with GelRed (Biotium, United States). The nucleic acids were visualized using Tanon 3500BR (Shanghai, China) and quantitatively analyzed using Quantity One software (Bio-Rad, United States). Two guides (FW-gDNA and RV-gDNA) were designed to target 100 bp regions (29% GC content) of the pUC19 plasmid for the plasmid cleavage assay. *Fp*Ago (400 nM), gDNA (2,000 nM), plasmid (1 μg), and 0.5 mM Mn^2+^ was mixed in reaction buffer. Reaction mixtures were incubated for 15 min at 75, 85, and 95°C. Samples were mixed with 5× loading buffer (Generay, Shanghai, China) before being resolved on 1% agarose gels. All nucleic acids used in this study are listed in Supplementary Tables 1, 2.

### Electrophoretic Mobility Shift Assay (EMSA)

*Fp*Ago was incubated with ssDNA guides (400 nM *Fp*Ago preloaded with 2,000 nM guides) in reaction buffer at 95°C for 15 min and then cooled to 25°C. After pre-incubation, 800 nM 45 nt FAM-labeled target DNA was added to a final 1:5:2 ratio (protein:guide:target) and incubated for 1 h at 37°C. Samples were then mixed with 10× loading buffer (50% [v/v] glycerol and 0.1% [w/v] bromophenol blue) and resolved by 8% native polyacrylamide gel electrophoresis (PAGE) with Tris-Borate-EDTA (TBE) buffer, at room temperature. Nucleic acids were visualized using a Tanon 3500BR microscope (Shanghai, China).

### Site-Directed Mutagenesis

Site-directed mutagenesis of the DEDD catalytic tetrad of *Fp*Ago to alanine was performed using a whole plasmid PCR method. The plasmid pET-28a (+)-*Fp*Ago was used as the initial template for PCR amplification using PrimeSTAR Max DNA Polymerase (Takara, Dalian, China), and mutations were introduced using the corresponding primers listed in Supplementary Table 3. The PCR products were then treated with *Dpn*I for an hour at 37°C, followed by direct transformation into competent *E. coli* BL21 (DE3) cells. The successful introduction of the desired mutants was confirmed by DNA sequencing (Genewiz, Inc., Suzhou, China).

### Homology Modeling and Structural Analysis of PAZ Domain of *Fp*Ago

The homology model of *Fp*Ago was performed with RosettaCM (Song et al., 2013) using the binary structure of *Mj*Ago with the 5′-P gDNA (PDB ID: 5G5T, identity 40.2%) as the template (Willkomm et al., 2017). A total of 1,000 models were built, and the best 10% of models by energy were identified and clustered. The PAZ domain alignments of *Fp*Ago were analyzed using PyMol software.

## Results

### Sequence Analysis of *Fp*Ago

**FIGURE 1:**
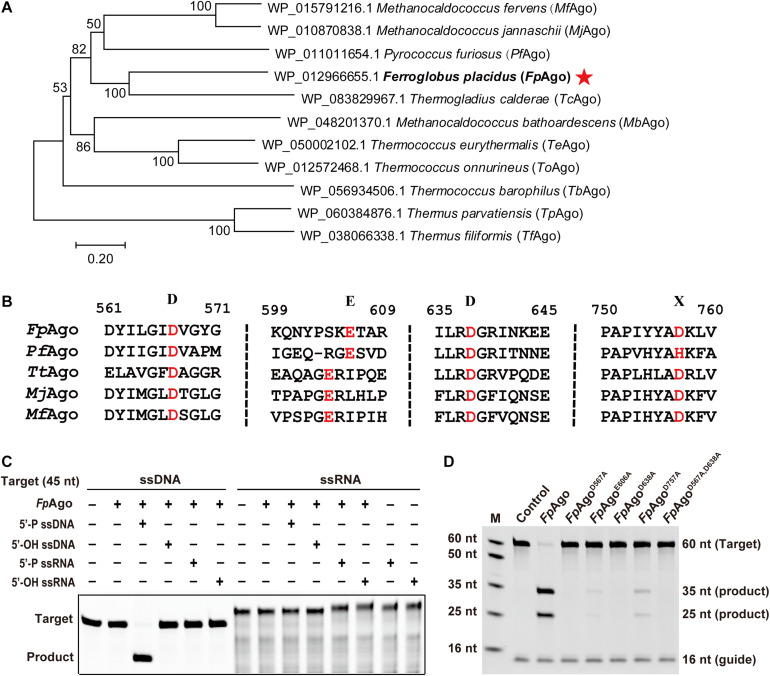
Phylogenetic analysis and cleavage activity assay of *Fp*Ago. **(A)** Neighbor-joining phylogenetic tree analysis of *Fp*Ago (marked by asterisks) based on amino acid sequences. The numbers at the nodes indicate the bootstrap values for neighbor-joining analysis of 1,000 resampled data sets. The scale bar represents the evolutionary distance between species. **(B)** Multiple sequence alignment of conserved amino acid residues (marked by red) of the DEDX tetrad localized in the PIWI domain of pAgos. **(C)** Guide and target preferences of *Fp*Ago. Cleavage activity assays of *Fp*Ago were performed with 45 nt FAM-labeled ssDNA or ssRNA target in reaction buffer containing 0.5 mM Mg^2+^ for 15 min at 95°C. **(D)** Cleavage activity of *Fp*Ago and its DEDD mutants. M, ssDNA marker; nt, nucleotide. The “Control” sample contains no protein.

To mine new functional resources in pAgos, we performed phylogenetic analysis by aligning the well-studied *Pf*Ago amino acid sequences in the NCBI database. Phylogenetic analysis revealed that in addition to two well-characterized hyperthermophilic pAgos, *Mj*Ago and *Mf*Ago, *Fp*Ago was most closely related to *Pf*Ago (Figure 1A). Multiple sequence alignments showed that *Fp*Ago has a conserved catalytic tetrad (DEDX) in the PIWI domain, indicating that *Fp*Ago may be catalytically active (Figure 1B).

### *Fp*Ago Mediates DNA-Guided DNA Cleavage

The recombinant *Fp*Ago and its mutants were heterologously expressed in *E. coli* BL21 (DE3), and the soluble fraction was purified and analyzed by SDS-PAGE. The purified protein showed a clear band, consistent with the predicted molecular weight of recombinant *Fp*Ago (94 kDa) (Supplementary Figure 1). Next, we tested the purified recombinant *Fp*Ago endonuclease activity using an *in vitro* cleavage assay. We used 16 nt RNA or DNA containing a 5′-P or 5′-OH group as guides to cleave complementary 45 nt FAM-labeled ssDNA or ssRNA as targets, respectively. The cleavage assays verified that *Fp*Ago does not show DNA-guided cleavage of RNA targets and only uses 5′-P guides to cleave the DNA target (Figure 1C). To experimentally identify the residues involved in *Fp*Ago activity, site-directed mutants were constructed. *Fp*Ago mutants D567A and D638A completely abolished *Fp*Ago activity, while E606A and D757A severely curtailed cleavage activity (Figure 1D), indicating that these four residues are the critical amino acid sites for *Fp*Ago activity.

### Effects of Temperature, Metal Ion, and NaCl on *Fp*Ago Cleavage Activity

**FIGURE 2:**
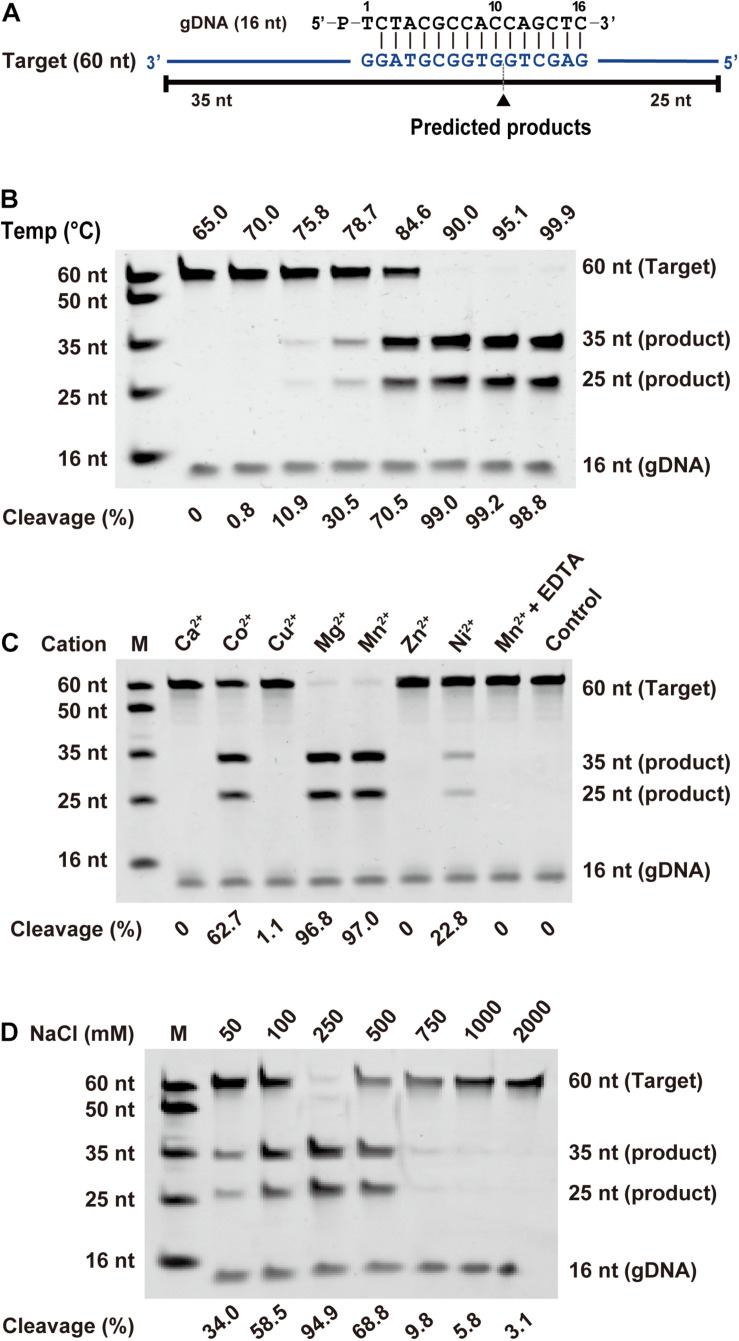
Effects of temperature, metal ion, and NaCl on *Fp*Ago cleavage activity. **(A)** Synthetic ssDNA guide (black) and target (blue). Predicted cleavage positions are indicated with a black triangle, black lines indicate the predicted 25 and 35 nt cleavage products from 60 nt target. **(B)** The optimal temperature of *Fp*Ago. **(C)** The divalent metal ions preferences of *Fp*Ago. Substrate cleavage was performed in the presence of different divalent metal ions and EDTA with a final concentration of 0.5 mM. **(D)** Effect of NaCl concentrations on the enzyme activity of *Fp*Ago. Cleavage experiments were carried out at the 1:5:2 molar ratio (400 nM *Fp*Ago: 2000 nM guide: 800 nM target) in reaction buffer containing 0.5 mM Mn^2+^ for 15 min at 95°C.

To investigate the biochemical properties of *Fp*Ago, we designed a 16 nt 5′-P gDNA to cleave a 60 nt ssDNA target (Figure 2A). We observed that the cleavage site at the target was located precisely between positions 10 and 11 from the 5′-end of the guide DNA, identical to all previously reported pAgo proteins. Considering that divalent metal ions are an essential co-factor for Agos activity, we investigated which divalent metal ions could support *Fp*Ago cleavage activity. We found that *Fp*Ago can widely utilize Mn^2+^, Mg^2+^, Co^2+^, and Ni^2+^ as cations, with the preference of Mn^2+^ > Mg^2+^ > Co^2+^ > Ni^2+^ (Figure 2C and Supplementary Figure 2). Here, we also observed that a high Mn^2+^ concentration (>2 mM) could inhibit the cleavage activity of *Fp*Ago, while *Fp*Ago decreased the activity to Mg^2+^ strength above 7 mM (Supplementary Figure 2). *Fp*Ago is sensitive to NaCl, with 250 mM as the optimal concentration, whereas a higher NaCl concentration reduced activity (Figure 2D), indicating that NaCl plays an important role in maintaining the catalytic activity and stability of *Fp*Ago.

### Effects of 5′-End Nucleotide and Modification of gDNA on Cleavage Activity

**FIGURE 3:**
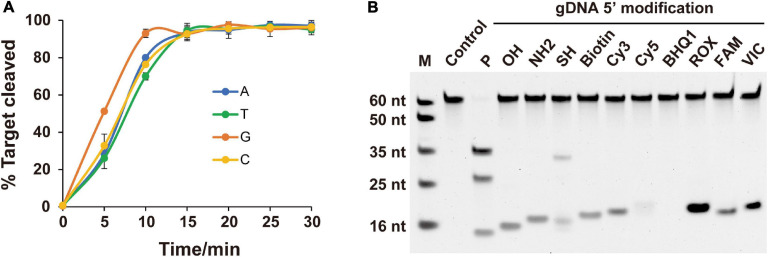
Effects of 5′-end nucleotides and modifications of gDNA on cleavage activity. **(A)** Cleavage kinetics of ssDNA target using 5′-P guided *Fp*Ago. Error bars represent the standard deviation of three independent experiments. **(B)** Effects of 5′-end modification of gDNA on *Fp*Ago cleavage activity.

As the preference for the 5′-end nucleotide of guides occurs in certain eAgos and pAgos (Lau et al., 2001; Frank et al., 2012; Willkomm et al., 2017), we tested whether *Fp*Ago prefers a specific 5′-end nucleotide on the gDNA. According to our time course cleavage assays, *Fp*Ago exhibited the highest initial reaction rate for 5′-dG at the 5′-end of the gDNA. Despite this preference, identical *Fp*Ago cleavage activities were observed after incubation for 15 min with gDNAs containing a 5′-end dC, dT, dA, or dG (Figure 3A). Unlike *Mj*Ago, which prefers a 5′-purine base of the gDNA (Willkomm et al., 2017), *Fp*Ago exhibits a preference specific for 5′-dG but not 5′-dA.

For most reported Agos, cleavage activity is strictly dependent on the presence of a 5′-P on the guide strand, although few Agos can use both 5′-P and 5′-OH guides for target cleavage (Cao et al., 2019; Chong et al., 2019; Kuzmenko et al., 2019). To determine the dependence of the 5′-end nucleotide modification on *Fp*Ago cleavage activity, we tested 11 different chemical modifications on the 5′-terminal of the guide DNA. The results demonstrated that 5′-P was required for *Fp*Ago cleavage activity (Figure 3B). When *Fp*Ago was loaded with 5′-SH gDNA, an uncanonical product band was observed on the gel. However, this brighter band likely resulted from the 5′-SH gDNA oxidation due to its instability under the tested conditions (Supplementary Figure 3).

### Effects of Target and gDNA Length on Cleavage Activity

**FIGURE 4:**
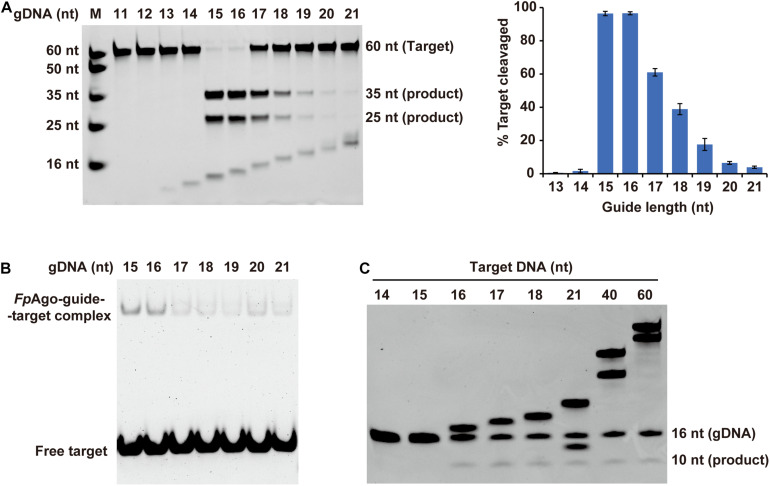
Effect of target and gDNA length on *Fp*Ago cleavage activity. **(A)** The gDNAs of 11–21 nt in lengths were tested for cleavage efficiency *in vitro*. Histogram data were calculated from gels quantitatively analyzed by Quantity One software. Error bars represent the SD of three independent experiments. **(B)** The affinities of *Fp*Ago form binary complexes with different length of 5′-P guide DNAs. **(C)** Minimum size of target used by *Fp*Ago. ssDNA targets of 14–18, 21, 40, and 60 nt in lengths were tested for cleavage efficiency *in vitro*. All cleavage experiments were carried out at the 1:5:2 molar ratio (400 nM *Fp*Ago: 2000 nM guide: 800 nM target) in reaction buffer containing 0.5 mM Mn^2+^ for 15 min at 95°C.

Previous structure-based mechanism studies on *Tt*Ago have demonstrated that for sufficient guide-target pairing, at least 15 bp DNA target formation, is required for *Tt*Ago switching from cleavage-incompatible to cleavage-compatible complexes (Sheng et al., 2014). In this study, the minimum guide length was studied. Strikingly, in contrast to previously reported eAgos and pAgos that function over a wide range of gDNA lengths between and 15–30 nt long, *Fp*Ago efficiently cleaves targets only with short gDNA of 15 and 16 nt length (Figure 4A). The optimal gDNA length was specific to 15–16 nt, and a more extended guide significantly decreased the cleavage efficiency. EMSA showed that the *Fp*Ago-guide-target ternary complex exhibited a stronger binding affinity for short 15 nt and 16 nt gDNAs (Figure 4B). In addition, we tested the target length to influence the cleavage. As expected, *Fp*Ago cut target DNAs with a minimal size of 16 nt. No cleavage activity loss was observed when the target was extended to 60 nt (Figure 4C).

### Effects of Mismatches on the Cleavage Activity of *Fp*Ago

**FIGURE 5:**
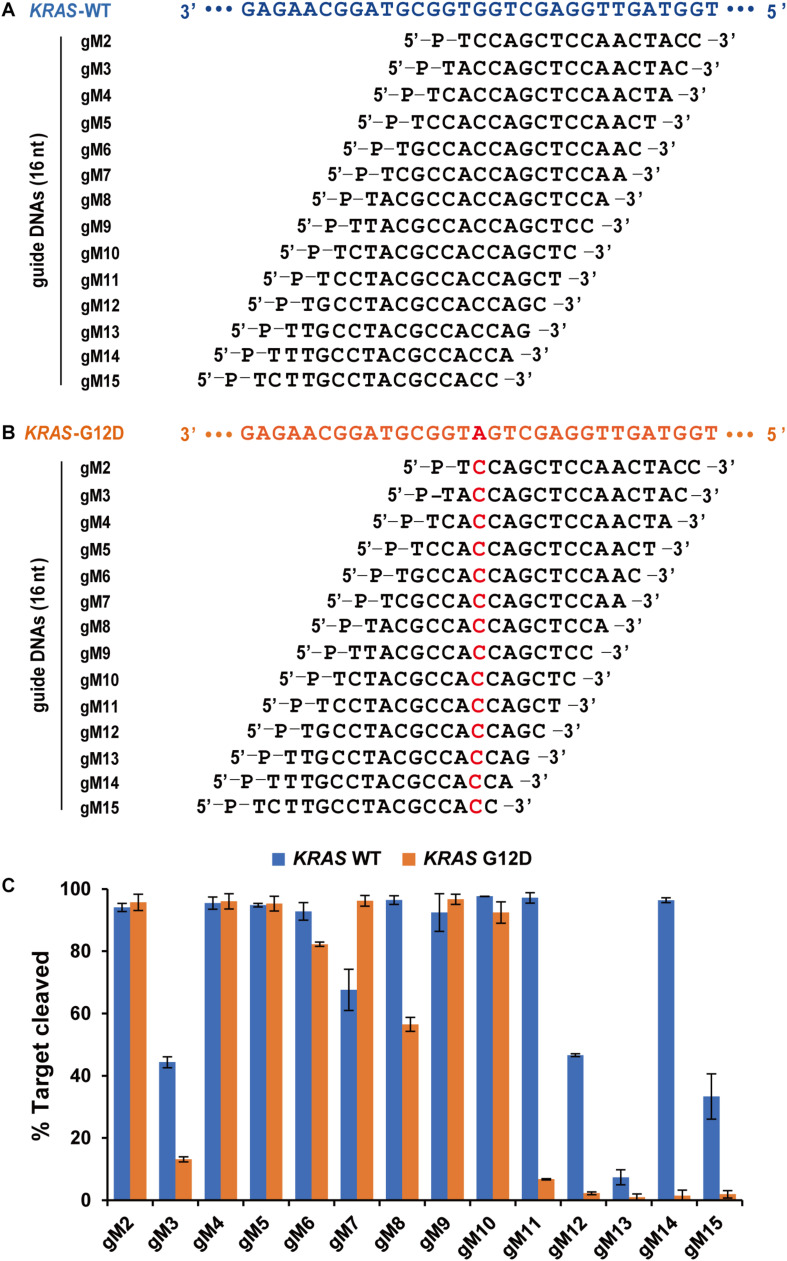
Effects of mismatches on the cleavage activity of *Fp*Ago. Sequences of designed guides corresponding to *KRAS* WT target **(A)** and mutant target **(B)**. All guides carry a base “T” as the first base based on the previous structural studies that 5′-end base of the guide strand does not participate in target pairing. **(C)** Cleavage efficiencies of WT *KRAS* DNA and *KRAS* G12D DNA mediated by guides. All cleavage experiments were carried out at the 1:5:2 molar ratio (400 nM *Fp*Ago: 2,000 nM guide: 800 nM target) in reaction buffer containing 0.5 mM Mg^2+^ for 15 min at 95°C.

Agos use small ssRNA or ssDNA guides to identify ssRNA or ssDNA targets through Watson-Crick base-pairing interactions, and mismatches between gDNA and target sequences may have significant effects on target recognition and cleavage (Wang et al., 2008; Schirle and MacRae, 2012; De et al., 2013). Previous studies have demonstrated that a single mismatch in the seed region of *Tt*Ago (positions 2–8) showed impaired cleavage activity (Wang et al., 2008). To investigate the effects of nucleotide mismatch in the guide-target duplex on *Fp*Ago activity, we introduced a single nucleotide mismatch at positions 2–15 in the 16 nt guide strand for cleavage of a *KRAS* G12D ssDNA target. The *KRAS* WT sequence was used as a control, paired completely with the above guides (Figures 5A,B). We observed that *Fp*Ago-mediated target cleavage was sensitive to single-base mismatches at positions 11–15 of gDNA. In contrast, mismatches in the seed region had little or no effect on the efficiency of target cleavage by *Fp*Ago. Notably, we observed that certain gDNAs, such as gM3, gM12, gM3, and gM15, resulted in lower cleavage efficiency even for the *KRAS* WT target. Surprisingly, a single mismatch at position 7 led to higher cleavage activity than that without mismatch in the guide-target duplex (Figure 5C).

### Homology Modeling and Structural Analysis of PAZ Domain of *Fp*Ago

**FIGURE 6:**
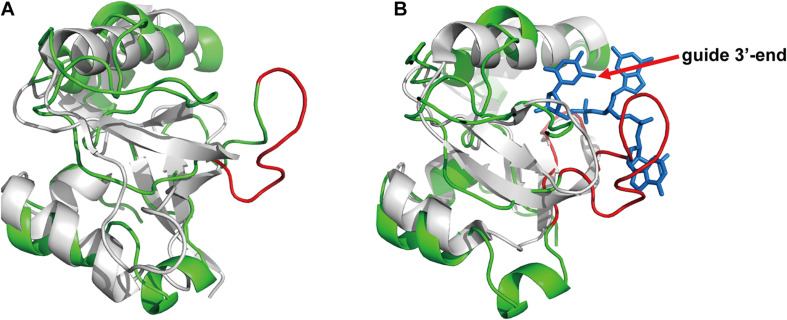
The PAZ domain comparison of the *F*pAgo with *Pf*Ago and *Mj*Ago via homology modeling. **(A)** The comparison of the *Fp*Ago (green) with apo *Pf*Ago (gray). **(B)** The comparison of the *Fp*Ago (green) with *Mj*Ago binary complex (gray) carrying a guide (blue). Only the last three nucleotides locating in the 3′-end of the guide strand were shown. A special loop employed by *Fp*Ago is highlighted in red.

Based on the above results on specific guide binding properties, we suggest that the unique structural conformation may guide the selection of guide length and mismatch tolerance of *Fp*Ago, especially for its interaction with the 3′-terminal of gDNA. Although we have tried to obtain the crystal structure, it is challenging to collect informatics data. Next, we performed homology modeling analysis to explain the structural details. Despite low primary sequence similarities, the overall fold of Agos is conserved in structure and consists of four canonical domains. Previous structural studies on Agos established that the 3′-end of the guide strand is anchored within the PAZ domain (Wang et al., 2008; Schirle and MacRae, 2012; De et al., 2013). Although *Fp*Ago has high sequence similarity with *Pf*Ago, *Fp*Ago has an extra loop compared to *Pf*Ago (Figure 6A). We further compared the PAZ domain between *Fp*Ago and the *Mj*Ago binary complex (*Mj*Ago carries a guide DNA strand). Interestingly, the stick-out loop in *Fp*Ago was vertically set in the middle of the guide 3′-end and PAZ pocket and likely narrowed the channel to bind the 3′-end of the guide (Figure 6B). We also performed sequence alignment analysis of *Fp*Ago PAZ domains with other Agos adopting long guides for cleavage activity. The results showed significant residue divergence in *Fp*Ago (Supplementary Figure 4), indicating that the *Fp*Ago PAZ domain has an additional conformation compared with other Agos. Thus, the unique extra loop employed by *Fp*Ago may explain the preference for *Fp*Ago to use shorter guides because longer guides are easily interfered with by the loop. This loop may also contribute to the sensitivity to mismatch at the 3′-end of the gDNA.

### *Fp*Ago Mediates Cleavage of Double-Stranded Plasmids

**FIGURE 7:**
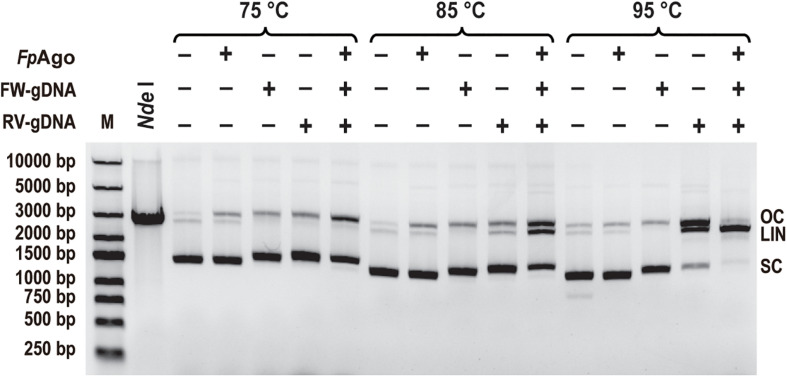
Plasmid cleavage by *Fp*Ago. *Fp*Ago-mediated plasmid cleavage after incubation for 15 min at different temperatures. *Nde*I lane: The extracted plasmid was digested with *Nde*I for 2 h, followed by 1% agarose gel electrophoresis. FW/RV-gDNA, forward and reverse guide DNA corresponded to a target site in plasmid pUC19; SC, supercoiled plasmid; LIN, linearized plasmid; OC, open circular plasmid.

To test whether *Fp*Ago cleaves plasmid DNA, *Fp*Ago was incubated with its pUC19 plasmid in the absence or presence of guides. Like *Pf*Ago, *Fp*Ago could nick plasmids in the absence of gDNA at 75 and 85°C, whereas the guide-independent plasmid nicking activity could be suppressed at 95°C (Figure 7). Guide-independent processing activity has also been observed for *Cb*Ago and *Km*Ago, and is suppressed at elevated temperatures (Kuzmenko et al., 2019; Liu et al., 2021b). When *Fp*Ago was loaded with a single gDNA, *Fp*Ago nicked or broke the plasmid DNA, generating open circular or linearized plasmids. The accumulation of linearized plasmids in the presence of RV-gDNAs but not FW-gDNAs may be because their efficiency is different, and the plasmid is unstable at elevated temperatures. When supplied with one pair of gDNAs, the efficiency of plasmid linearization was increased, in particular, by increasing the temperature of the reaction (Figure 7), which likely facilitated plasmid DNA melting and enhanced *Fp*Ago activity at elevated temperatures. These results demonstrate that *Fp*Ago could efficiently mediate DNA-guided cleavage of dsDNA plasmids if both strands of the plasmid DNA are targeted.

## Discussion

### *Fp*Ago Can Cleave Both ssDNA and dsDNA Target Directed by gDNA

Prokaryotic Argonaute proteins has been shown to have more functional and mechanistic diversity than eAgos (Swarts et al., 2014b; Hegge et al., 2018). Previous attempts have been made to discover pAgos with genome-editing capabilities, and some pAgos from mesophilic bacteria have been described. The cleavage of double-stranded DNA (dsDNA) mediated by mesophilic Agos is likely dependent on the local sequence context because the Agos lack DNA helicase activity (Hegge et al., 2018, 2019; Cao et al., 2019; Kuzmenko et al., 2019; Liu et al., 2021b). Therefore, increasing substrate accessibility when using mesophilic pAgos in biotechnology is regarded as a key challenge. The dsDNA cleavage by hyperthermophilic Agos is considered as two independent processes of ssDNA nicks since dsDNA denatures at elevated temperatures (Swarts et al., 2015). Here, we describe a novel *Fp*Ago from the hyperthermophilic *F. placidus*. *Fp*Ago functions as a DNA-guided DNA endonuclease that requires divalent metal ions such as Mn^2+^, Mg^2+^, Co^2+^, or Ni^2+^ for its activity. Although *Fp*Ago prefers Mn^2+^ over Mg^2+^ for cleavage, similar to most Agos, *Fp*Ago displays a higher tolerance for Mg^2+^ over Mn^2+^. Moreover, *Fp*Ago efficiently cleaves double-stranded plasmids at elevated temperatures, demonstrating its potential application in dsDNA manipulation. To best of knowledge, specific DNA helicase can enhance thermophilic TtAgos activity by strand invasion and unwinding of dsDNA (Hunt et al., 2018). And no putative DNA helicase in proximity (within 5 kb of *Fp*Ago) was found in the *F. placidus* genome via bioinformatic analysis. We proposed the optimal growing temperature for *F. placidus*, which would be sufficient for the unwinding dsDNA without the requirement of helicase.

### *Fp*Ago Exhibits a Preference for a 5′-dG Base at the 5′ End of the gDNA

A previous analysis of the nucleotide composition of ssDNA sequences co-purified with *Tt*Ago revealed that *Tt*Ago has a strong bias for a 5′-end deoxycytidine. However, *in vitro* cleavage assays showed that all guides containing a 5′-dC, dT, dA, or dG resulted in similar target cleavage activities (Swarts et al., 2014a). The 5′-end base of the guide strand and 1st base of the target splayed out in separate binding pockets, respectively, was observed in the crystal structure of *Tt*Ago (Sheng et al., 2014). The identical target cleavage activities could be explained by the fact that the 5′-end base of the guide strand does not participate in target pairing. Interestingly, *Mj*Ago-mediated cleavage efficiency is significantly higher using 5′-purine guides than 5′-pyrimidines guides (Willkomm et al., 2017). For *Fp*Ago, we found that a guide with a 5′-dG supports higher cleavage efficiency than the other three types of guides. Earlier findings imply that the higher affinity of 5′-dG ternary *Fp*Ago-guide complexes resulted in the increase of cleavage efficiency by increasing the number of ternary *Fp*Ago-guide-target complexes compared to other guides (Deerberg et al., 2013; Willkomm et al., 2016, 2017). A moderate AT-bias near the 5′-end and downstream of the target cleavage site was observed for mesophilic *Cb*Ago when expressed and purified from *E*. coli cells, indicating that their biogenesis might depend on the melting of these DNA regions in the context of dsDNA (Kuzmenko et al., 2020). The 5′-dC preference of thermophilic *Tt*Ago *in vivo* was thought to be the result of specific guide processing or preferential 5′ nucleoside selection by *Tt*Ago (Swarts et al., 2014a). Taken together, the 5′-dG has a preference for hyperthermophilic *Fp*Ago, suggesting that the efficiency of gDNA processing does not strongly depend on the GC content, especially for thermophilic pAgos.

### *Fp*Ago Tolerates Mismatches in the Seed Region but Is Sensitive to Mismatches in the 3′-Terminal of gDNA

It is generally acknowledged that even a single mismatch in the seed region reduces cleavage activity, whereas mismatches in the 3′-guide strands are usually tolerated without significant loss of cleavage activity (Dahlgren et al., 2008; Wang et al., 2008; Parker et al., 2009; Salomon et al., 2015). In this study, we observed that mismatches in the seed region did not significantly affect *Fp*Ago cleavage activity, and conversely, even stimulated target DNA cleavage (for gM7). Coincidentally, a recent study on zebrafish Argonaute2 demonstrated that a mismatch involving guide-RNA position 6 enhances slicing activity, whereas, without the mismatch, cleavage is essentially abolished (Chen et al., 2017). Together, these findings suggest that some seed mismatches have opposite effects on cleavage activity. In contrast, for mismatches in the 3′-guide strand, target cleavage was either almost abolished or strongly reduced by *Fp*Ago, and similar effects were recently reported for *Tt*Ago, *Cb*Ago, and *Lr*Ago (Kuzmenko et al., 2019; Song et al., 2020). Surprisingly, *Fp*Ago was less active with a perfectly complementary target mediated by gDNAs of gM3, gM12, gM3, and gM15. These results suggest that the cleavage activity of *Fp*Ago depends on the sequences of the guide and target.

### gDNA Recognition by the Distinctive *Fp*Ago PAZ Domain

Both eAgos and long pAgos generally consist of four conserved structure domain architectures, namely, MID (middle) domain, PIWI (P-body-induced wimpy testes) domain, PAZ (PIWI-Argonaute-Zwille) domain, and N domain. The MID and PAZ domains usually form binding pockets that anchor the 5′- and 3′-ends of the guide strand, respectively. The PIWI domain contains a catalytic motif that mediates target strand cleavage (Schirle and MacRae, 2012; Hegge et al., 2018). Ago-mediated cleavage of targets by guide strands up to 36 nt in length could be rationalized by the guide to adopting an alternative trajectory to allow 3′-end insertion into the PAZ binding pocket. In addition, the open nucleic acid-binding channel, between the PAZ domain and PIWI domain, which encompasses the 3′-end of the guide strand, is accessible to the outside and permissive to looping-out longer 3′-end (Wang et al., 2008). *Fp*Ago adopts a shorter guide strand for cleavage activity, indicating a distinct catalytic pattern. Previous structural studies based on *Tt*Ago showed that the retained or released state of the 3′-end of the guide strand within the PAZ binding pocket transforms the *Tt*Ago ternary complex from a cleavage-incompatible conformation to a cleavage-compatible conformation (Sheng et al., 2014). Our structural analysis indicates that the severely reduced activity mediated by long guide strands may be explained by the fact that *Fp*Ago employs a distinct PAZ domain that restricts the 3′-ends of the long guide strand inserted into the PAZ binding pocket, which may result in the instability of guide-target duplex hybridization. Our EMSA assays also showed that the *Fp*Ago-guide-target ternary complex exhibited a weaker binding affinity for long guide strands. Indeed, structural work on *Fp*Ago and its binary and ternary complexes is needed to elucidate the molecular basis for the short guide strand in the future.

## Conclusion

This study identified a novel hyperthermophilic *Fp*Ago that cleaves DNA targets in a 5′-P gDNA-dependent sequence-specific manner. Starting from a minimal gDNA length of 15 nt, *Fp*Ago only accepted gDNA lengths of less than 21 nt. *Fp*Ago prefers the 5′-dG of the guide strand. The single mismatch near the 3′-terminal of gDNA rather than the seed region curtails the *Fp*Ago cleavage activity. The structure modeling analysis implicates a unique loop of the PAZ domain that may be responsible for the specificity of *Fp*Ago for guide binding. Furthermore, *Fp*Ago efficiently mediates DNA-guided DNA cleavage of double-stranded plasmids at elevated temperatures. These findings broaden our understanding of thermophilic Agos and pave the way for their use in DNA manipulations in the future.

## Data Availability Statement

Publicly available datasets were analyzed in this study. This data can be found here: National Center for Biotechnology Information (NCBI) Protein, https://www.ncbi.nlm.nih.gov/protein/, WP_012966655.1.

## Author Contributions

XG performed the research, data analysis, and preparation of the manuscript. YF and QL provided guidance, designed the experiments, and revised the manuscript. YS assisted with cleavage assays and data analysis. LC and FH performed structural modeling analysis. All authors participated in the project proposal and approved the final version of the manuscript.

## Conflict of Interest

The authors declare that the research was conducted in the absence of any commercial or financial relationships that could be construed as a potential conflict of interest.
