# Supplementary material for: A Hyperthermophilic Argonaute From Ferroglobus placidus With Specificity on Guide Binding Pattern
Source: Front Microbiol. 2021 Jun 9;12:654345. doi: 10.3389/fmicb.2021.654345 (PMC8248672; doi:10.3389/fmicb.2021.654345)
Supplement: Supplementary file 1 [file Data_Sheet_1.PDF]

Supplementary Information

A hyperthermophilic Argonaute from *Ferroglobus placidus* with specificity on guide binding pattern

Xiang Guo[1], Yingying Sun[1], Liuqing Chen[2], Fei Huang[1], Qian Liu[1*], Yan Feng[1]*

[1]State Key Laboratory of Microbial Metabolism, School of Life Sciences and Biotechnology, Shanghai Jiao Tong University, Shanghai 200240, China

[2]Shenzhen Institutes of Advanced Technology, Chinese Academy of Sciences, Shenzhen 518055, China

*** Correspondence:**
To whom correspondence should be addressed. Tel: +86-021-34207189; Fax: +86-021-34207189;
Email: liuqian1018@sjtu.edu.cn, yfeng2009@sjtu.edu.cn

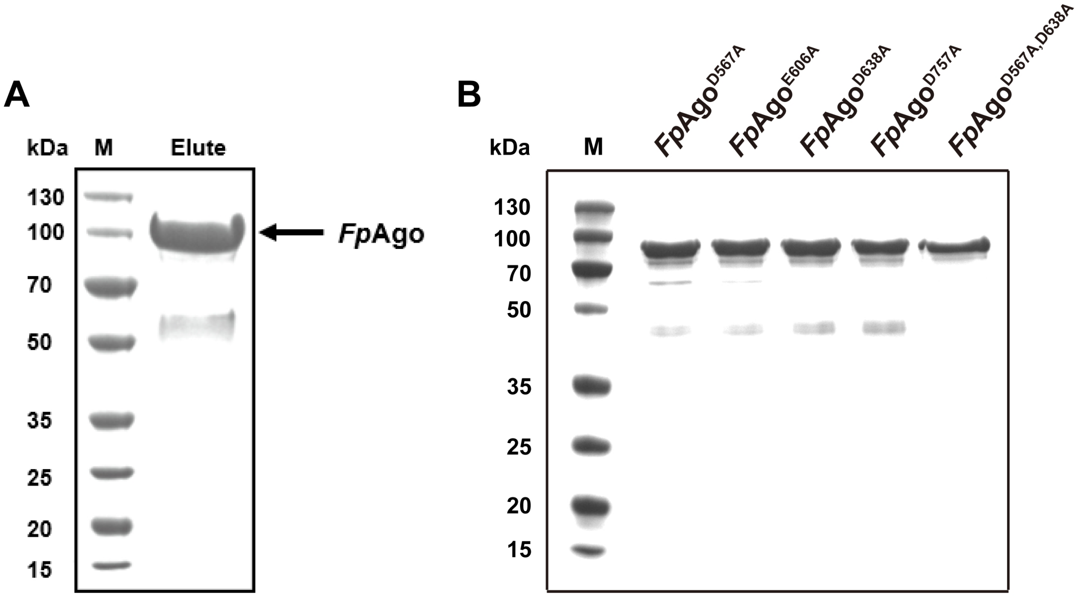

FIGURE S1 SDS-PAGE analysis of *Fp*Ago **(A)** and its mutants **(B)** purified by Ni-NTA-affinity chromatography.

A

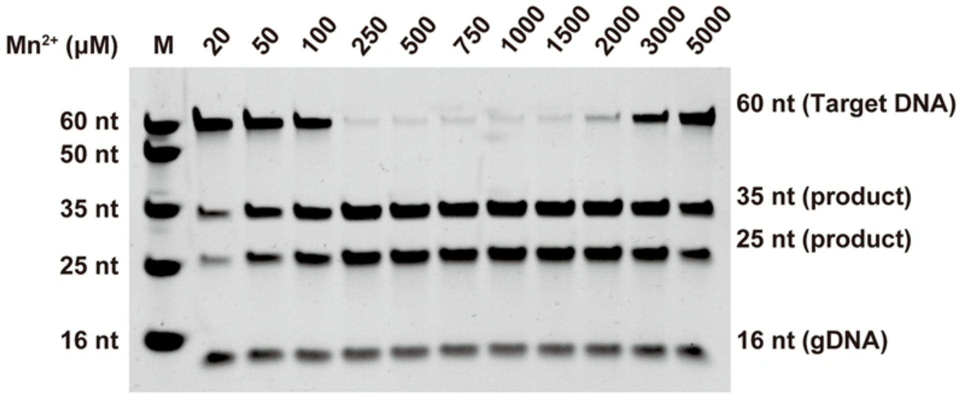

B

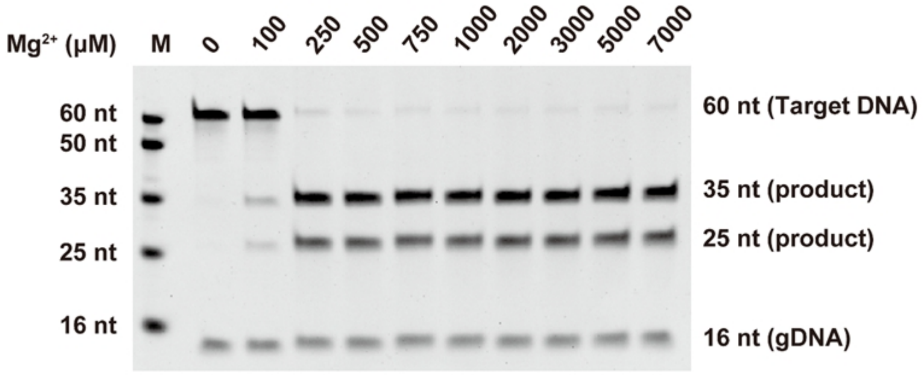

FIGURE S2 Effects of metal ions concentrations on *Fp*Ago activity. **(A)** Effect of Mn^{2+} concentrations on *Fp*Ago activity. **(B)** Effect of Mg^{2+} concentrations on *Fp*Ago activity.

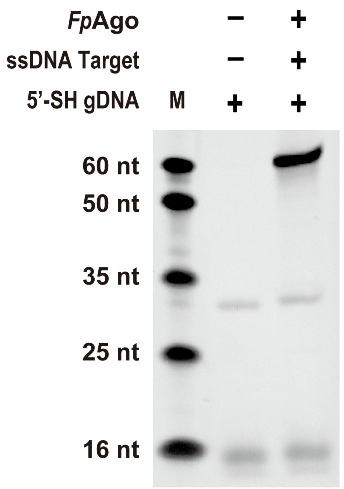

FIGURE S3 Evaluation of *Fp*Ago cleavage activity in the presence of 5'-SH gDNA.

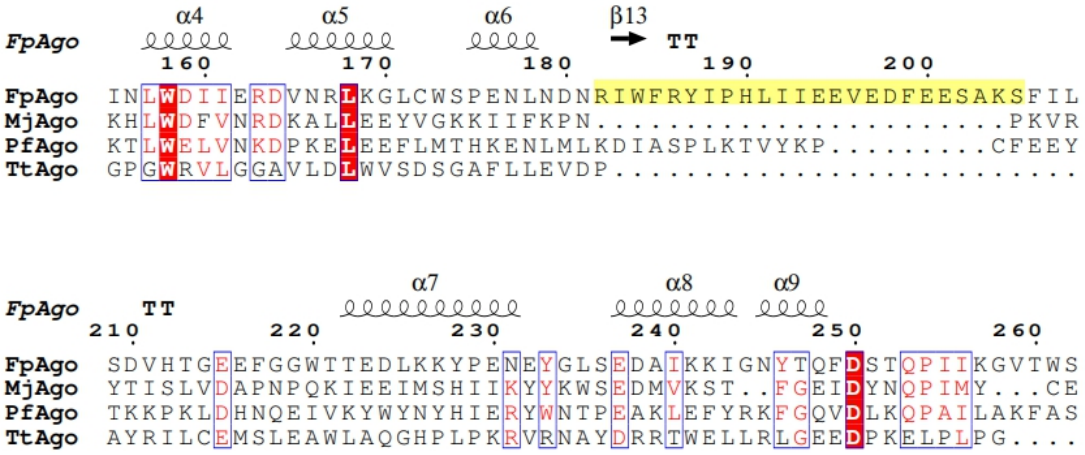

FIGURE S4 Structure-based sequence alignments of PAZ domains of Ago proteins. Special residues of *Fp*Ago highlighted in yellow.

Table S1 Target DNA used in this study.

Name	Target sequence (5'-3')	Presence
45 nt-ssDNA (3' FAM)	GGTAGTTGGAGCTGGTGGCGTA GGCAAGAGTGCCTTGACGATACA	Figs. 1,4
45 nt-ssRNA (3' FAM)	GGUAGUUGGAGCUGGUGGCGUA GGCAAGAGUGCCUUGACGAUACA	Fig. 1
KRAS-WT	AGCTTACTTGTGGTAGTTGGAGCT GGTGGCGTAGGCAAGAGTGCCTT GACGATACAGCTA	Figs. 2-5
KRAS-G12D	AGCTTACTTGTGGTAGTTGGAGCT GATGGCGTAGGCAAGAGTGCCTT GACGATACAGCTA	Fig. 5
14 nt target	GCTGGTGGCGTAGA	Fig. 4
15 nt target	AGCTGGTGGCGTAGA	Fig. 4
16 nt target	GAGCTGGTGGCGTAGA	Fig. 4
17 nt target	GGAGCTGGTGGCGTAGA	Fig. 4
18 nt target	TGGAGCTGGTGGCGTAGA	Fig. 4
21 nt target	AGTTGGAGCTGGTGGCGTAGA	Fig. 4
40 nt target	CTGAAAGCTTACTTGTGGTAGTT GGAGCTGGTGGCGTAGA	Fig. 4
60 nt target	ATAAGGCCTGCTGAAAATGACTG AAAGCTTACTTGTGGTAGTTGGA GCTGGTGGCGTAGA	Fig. 4
pUC19 plasmid targeted 100 bp sequence	GAACGAAAACTCACGTTAAGGGA TTTTGGTCATGAGATTATCAAAAA GGATCTTCACCTAGATCCTTTTAA ATTAAAAATGAAGTTTTAAATCA ATCTAA	Fig. 7

Table S2 gDNA used in this study.

Name	gDNA sequence (5’-3’)	Presence
16 nt-gDNA (5’-P)	P-TCTACGCCACCAGCTC	Fig. 1
16 nt-gDNA (5’-OH)	OH-TCTACGCCACCAGCTC	Fig. 1
16 nt-gRNA (5’-P)	P-UCUACGCCACCAGCUC	Fig. 1
16 nt-gRNA (5’-OH)	OH-UCUACGCCACCAGCUC	Fig. 1
gDNA (5’-NH_2C_6)	NH_2C_6-TCTACGCCACCAGCTC	Fig. 3
gDNA (5’-SHC_6)	SHC_6-TCTACGCCACCAGCTC	Fig. 3
gDNA (5’-Biotin)	Biotin-TCTACGCCACCAGCTC	Fig. 3
gDNA (5’-FAM)	FAM-TCTACGCCACCAGCTC	Fig. 3
gDNA (5’-VIC)	VIC-TCTACGCCACCAGCTC	Fig. 3
gDNA (5’-ROX)	ROX-TCTACGCCACCAGCTC	Fig. 3
gDNA (5’-Cy3)	Cy3-TCTACGCCACCAGCTC	Fig. 3
gDNA (5’-Cy5)	Cy5-TCTACGCCACCAGCTC	Fig. 3
gDNA (5’-BHQ1)	BHQ1-TCTACGCCACCAGCTC	Fig. 3
gDNA-11 nt	P-TCTACGCCACC	Fig. 4
gDNA-12 nt	P-TCTACGCCACCA	Fig. 4
gDNA-13 nt	P-TCTACGCCACCAG	Fig. 4
gDNA-14 nt	P-TCTACGCCACCAGC	Fig. 4
gDNA-15 nt	P-TCTACGCCACCAGCT	Fig. 4
gDNA-17 nt	P-TCTACGCCACCAGCTCC	Fig. 4
gDNA-18 nt	P-TCTACGCCACCAGCTCCA	Fig. 4
gDNA-19 nt	P-TCTACGCCACCAGCTCCAA	Fig. 4
gDNA-20 nt	P-TCTACGCCACCAGCTCCAAC	Fig. 4
gDNA-21 nt	P-TCTACGCCACCAGCTCCAACT	Fig. 4
gDNA-MP2	P-TCCAGCTCCAACTACC	Fig. 5
gDNA-MP3	P-TACCAGCTCCAACTAC	Fig. 5
gDNA-MP4	P-TCACCAGCTCCAACTA	Fig. 5
gDNA-MP5	P-TCCACCAGCTCCAACT	Fig. 5
gDNA-MP6	P-TGCCACCAGCTCCAAC	Fig. 5
gDNA-MP7	P-TCGCCACCAGCTCCAA	Fig. 5
gDNA-MP8	P-TACGCCACCAGCTCCA	Fig. 5
gDNA-MP9	P-TTACGCCACCAGCTCC	Fig. 5
gDNA-MP10	P-TCTACGCCACCAGCTC	Fig. 5
gDNA-MP11	P-TCCTACGCCACCAGCT	Fig. 5
gDNA-MP12	P-TGCCTACGCCACCAGC	Fig. 5
gDNA-MP13	P-TTGCCTACGCCACCAG	Fig. 5
gDNA-MP14	P-TTTGCCTACGCCACCA	Fig. 5
gDNA-MP15	P-TCTTGCCTACGCCACC	Fig. 5
FW-gDNA	P-TCAAAAAGGATCTTCA	Fig. 7
RV-gDNA	P-TAGGTGAAGATCCTTT	Fig. 7

Table S3 Mutation primers used in this study.

Name	Primer sequence (5’-3’)	Presence
D567A FW	GATTACATTCTGGGTATCGC GGTAGGCTACGGCGAAGCG	Fig. 1
D567A RV	CGCTTCGCCGTAGCCTACCG CGATACCCAGAATGTAATC	Fig. 1
E606A FW	GAACTACCCATCTAAAGCG ACCGCGCGTATTAAAG	Fig. 1
E606A RV	CTTTAATACGCGCGGTCGC TTTAGATGGGTAGTTC	Fig. 1
D638A FW	CTATCCTGATCCTGCGTGCG GGCCGTATCAACAAAGAAG	Fig. 1
D638A RV	CTTCTTTGTTGATACGGCCC GCACGCAGGATCAGGATAG	Fig. 1
D757A FW	CGATCTACTACGCGGCGAA GCTGGTTAAAGC	Fig. 1
D757A RV	GCTTTAACCAGCTTCGCCG CGTAGTAGATCG	Fig. 1
